# Intraspecific divergence in sperm morphology of the green sea urchin, *Strongylocentrotus droebachiensis*: implications for selection in broadcast spawners

**DOI:** 10.1186/1471-2148-8-283

**Published:** 2008-10-13

**Authors:** Mollie K Manier, Stephen R Palumbi

**Affiliations:** 1 Department of Biological Sciences, Hopkins Marine Station, Stanford University, Pacific Grove, CA, USA; 2 Current address: Department of Biology, 110 Life Sciences Complex, Syracuse University, Syracuse, NY 13244, USA

## Abstract

**Background:**

Sperm morphology can be highly variable among species, but less is known about patterns of population differentiation within species. Most studies of sperm morphometric variation are done in species with internal fertilization, where sexual selection can be mediated by complex mating behavior and the environment of the female reproductive tract. Far less is known about patterns of sperm evolution in broadcast spawners, where reproductive dynamics are largely carried out at the gametic level. We investigated variation in sperm morphology of a broadcast spawner, the green sea urchin (*Strongylocentrotus droebachiensis*), within and among spawnings of an individual, among individuals within a population, and among populations. We also examined population-level variation between two reproductive seasons for one population. We then compared among-population quantitative genetic divergence (*Q*_ST_) for sperm characters to divergence at neutral microsatellite markers (*F*_ST_).

**Results:**

All sperm traits except total length showed strong patterns of high diversity among populations, as did overall sperm morphology quantified using multivariate analysis. We also found significant differences in almost all traits among individuals in all populations. Head length, axoneme length, and total length had high within-male repeatability across multiple spawnings. Only sperm head width had significant within-population variation across two reproductive seasons. We found signatures of directional selection on head length and head width, with strong selection possibly acting on head length between the Pacific and West Atlantic populations. We also discuss the strengths and limitations of the *Q*_ST_-*F*_ST _comparison.

**Conclusion:**

Sperm morphology in *S. droebachiensis *is highly variable, both among populations and among individuals within populations, and has low variation within an individual across multiple spawnings. Selective pressures acting among populations may differ from those acting within, with directional selection implicated in driving divergence among populations and balancing selection as a possible mechanism for producing variability among males. Sexual selection in broadcast spawners may be mediated by different processes from those acting on internal fertilizers. Selective divergence in sperm head length among populations is associated with ecological differences among populations that may play a large role in mediating sexual selection in this broadcast spawner.

## Background

Spermatozoa are the most morphologically diverse cells, yet they all have the same basic function: to fertilize an egg. Variation in sperm shape among species can often be attributed to sexual selection mediated by sperm competition [1-8] (but see [9]), but variation within species has been more difficult to explain. Male-to-male variation in sperm morphology has been documented in many species spanning a wide range of mating systems [10,11]. Functional studies investigating the within-species association between sperm length and fertilization success have found an advantage of larger sperm [12-16], but sometimes the race goes to the short [17]. Most often, though, no evidence is found for an intraspecific association of sperm morphology and sperm competition [18-27]. Thus, it remains unknown if selection is acting on sperm morphology in a similar way within and between species, or if sperm are evolving under complex, possibly context-dependent selective regimes that vary among species and populations [16,17,28].

Most studies of sperm variation focus on organisms with internal fertilization, where sperm act in the context of an environment controlled by the female reproductive tract [10,16,17]. In such cases, sperm competition is dependent on multiple matings by the female and may be influenced by efforts of males to displace sperm from prior matings or to guard females against subsequent matings [29]. There may also be indirect effects of males on female behavior [30].

Some external fertilizers can likewise have complex mating systems with mate choice, male-male competition, and alternative male mating strategies [7,21,26]. However, fertilization dynamics of broadcast spawning organisms may face different selective rules. Mating occurs when typically sessile or sedentary adults release their gametes into the environment, resulting in external fertilization, and has been best studied in marine systems. Some species, particularly invertebrates, have a limited behavioral repertoire as adults and exhibit little behavioral mate choice. In dioecious species, female multiple mating is controlled largely by the density of males in her vicinity, and competition between males is relegated to the level of the gamete. Sperm competition may be high when synchronous spawning occurs in dense aggregations [31,32], but intensity of sperm competition (as a function of sperm density), and therefore sexual selection, may vary over small spatial and temporal scales with changes in population density. Furthermore, fertilization conditions may be additionally influenced by variation in factors including wave action [33,34], temperature [35-37], or egg size [34,38], creating a selective mosaic in which no single sperm type is universally preferred. Sperm variation among species of broadcast spawners is well-described [39-42] and can be associated with egg size and developmental mode [40]. While little is known about evolutionary forces acting on sperm morphology within a broadcast spawning species, they are certainly more related to ecological environmental variation rather than to conditions within a female reproductive tract.

A basic question is whether sperm morphology of a broadcast spawner varies substantially among males, as has been found in both internal and other external fertilizers [10,11]. Additionally, does sperm morphology vary among populations, a pattern that precedes species-level divergence? We address these questions in the green sea urchin (*Strongylocentrotus droebachiensis*), by examining variation in five sperm morphometrics within and among Pacific, West Atlantic and East Atlantic populations. We also assess the stability of sperm parameters over time within an individual across multiple spawnings and within a population across two reproductive seasons.

In order to determine if selection is driving population-level divergence in sperm morphology, we compare quantitative genetic divergence in sperm traits (*Q*_ST_) with a neutral expectation of differentiation under genetic drift, estimated by divergence at neutral microsatellite loci (*F*_ST _[43-46]). The comparison of *Q*_ST _with *F*_ST _is a useful tool for identifying local adaptive differentiation in fitness-related quantitative traits, because it allows us to test a hypothesis of selection against three predictions [44]. If sperm morphology is neutrally divergent among populations, we would expect to see comparable patterns of variation at both sperm morphometrics and neutral microsatellites (*Q*_ST _= *F*_ST_). If sperm traits are under directional selection for different optima among populations, quantitative trait divergence should be higher than expected under neutrality (*Q*_ST _> *F*_ST_). Finally, if sperm traits are evolving under homogenizing selection, population means should be more similar than expected under neutrality (*Q*_ST _<*F*_ST_), though this conclusion is much more difficult to obtain with confidence. *Q*_ST_-*F*_ST _analysis has been applied in a wide range of taxa to address diverse questions in evolutionary biology, e.g., [47-49].

Here, we show that sperm traits have diverged strongly among populations as well as among individuals within populations. At the same time, sperm morphology exhibits low variation within individual males across multiple spawnings. We detect directional selection on sperm traits for different population means, especially in sperm head length between the Pacific and West Atlantic populations. Patterns of pairwise divergence among populations suggests that ecological variables may be playing a large role in sperm evolution of this broadcast spawner.

## Results and Discussion

### Variation within males

**Figure 1:**
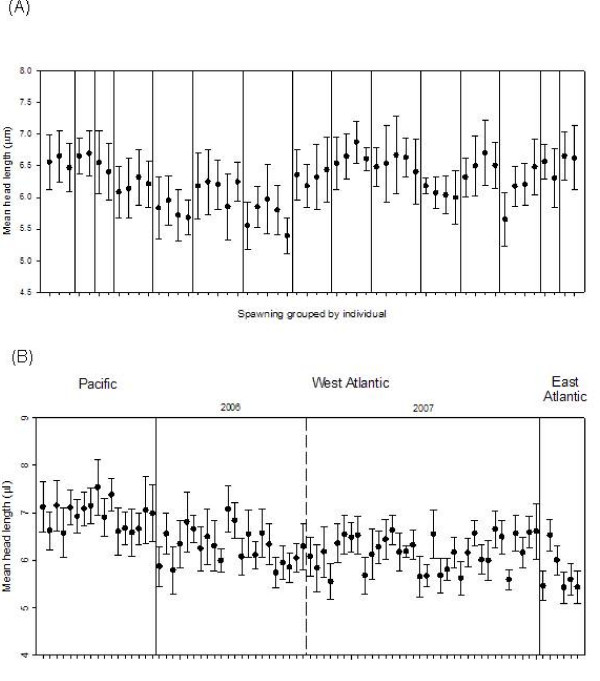
**Sperm head length variation**. Mean individual sperm head length (error bars ± 1 SD) (A) among spawnings within individuals (separated by vertical solid lines). (B) within and among populations (separated by vertical solid lines), and 2006 and 2007 samples from the West Atlantic (separated by vertical dashed line).

We tested sperm from multiple spawnings of males held in culture in 2006 and 2007. Among 15 males measured every two weeks two to five times (average = 3), head length, axoneme length and total length did not differ significantly among spawning events (Table 1; for brevity, only head length shown in Figure 1A). However, head width and midpiece area showed significant within-individual variation over time. Repeatability ranged from 0.2 to 0.77, with the highest repeatabilities obtained for head length, axoneme length and total length (Table 2). These repeatabilities are estimates of the upper limit of broad-sense heritability and were used in the calculation of *Q*_ST _for each trait.

**Table 1:** Summary of results testing for differences between various groups.

	Measure		Year		Spawn		Pacific		West Atlantic		East Atlantic		Pop	
	*F*	*P*	*t*	*P*	*F*	*P*	*F*	*P*	*F*	*P*	*F*	*P*	*F*	*P*
HL	0.55	0.46	1.22	0.2287	2.32	0.09777	8.27	<0.0001	11.23	<0.0001	44.47	<0.0001	36.48	<0.0001
HW	0.46	0.50	3.88	0.0005	7.31	0.00097	5.4	<0.0001	25.76, 9.13	<0.0001, <0.0001	15.82	<0.0001	23.61	<0.0001
AL	0.04	0.84	-0.56	0.5765	0.61	0.61723	8.86	<0.0001	10.98	<0.0001	4.51	0.0008	8.43	0.0005
TOTAL	0.00	0.98	-0.53	0.598	0.95	0.42989	6.79	<0.0001	10.36	<0.0001	4.4	0.001	2.9	0.0612
MA	3.62	0.06	2.45	0.0194	4.84	0.008	13.23	<0.0001	12.5	<0.0001	0.76	0.5789	8.15	<0.0001

Results for trait differences between measurement events (Measure), 2006 and 2007 West Atlantic sperm traits (Year), among spawnings within an individual (Spawn), among individuals within populations (Pacific, West Atlantic, East Atlantic), and among populations (Pop). For HW, individual differences within West Atlantic reported for 2006 and 2007, respectively, and Pop reflects separate accounting for the West Atlantic population by year. Sperm traits are head length (HL), head width (HW), axoneme length (AL), total length (TOTAL), midpiece area (MA).

**Table 2:** Results of global *Q*_ST _analysis for sperm traits.

	Repeatability	*Q*_ST_	*F*	*P*	*Q*_ST_/*F*_ST_	*P*_*X*_^2^
HL	0.77	0.57	36.48	< 0.0001	3.57	0.0283
HW	0.29	0.65	20.09	< 0.0001	4.10	0.0166
AL	0.58	0.27	8.43	0.0005	1.68	0.1865
TOTAL	0.56	0.09	2.90	0.0612	0.55	0.5755
MA	0.20	0.50	8.03	0.0007	3.14	0.0432
Average	0.48	0.41				
SE	0.10	0.10				

Microsatellite-based *F*_ST _= 0.159. *F *and *P *correspond to the ANOVA from which variance components were obtained for calculating *Q*_ST_. *P*_*X*_^2 ^is the p-value associated with the chi-squared distribution, for the test statistic (*n*_demes _- 1)*Q*_ST_/*F*_ST_.

Within-male variability in sperm morphometry as measured by within-male CV did not differ for the three populations for any trait except head width (*F *= 8.92; *P *< 0.0001). CV of head length among males within a population showed a clinal pattern with lowest among-male variability in the Pacific and highest in the East Atlantic. In all other traits, the West Atlantic had the highest variability among males, with the lowest in Norway. Head width was an exception, with the lowest variability among males in the Pacific. In general, patterns of variability within and among individuals were comparable for all traits and all populations, but midpiece area in the East Atlantic was an order of magnitude more variable within males than among males. In fact, midpiece area in all populations was much more variable than the other traits, with CV's within males of 47.8 in the Pacific, 62.1 in the West Atlantic and 31.7 in the East Atlantic. Among-male CV's for midpiece area were 33.1 in the Pacific, 42.5 in the West Atlantic, and 5.6 in the East Atlantic.

In our study, we found strong evidence that sperm morphology is controlled more by developmental factors during spermatogenesis than ambient environmental conditions. We examined repeatability for West Atlantic males that were removed from the field and placed into a common laboratory environment. All sea urchins were spawned upon arrival from the field, so the first spawning should reflect sperm morphology under the native ecological conditions of the population. Individuals were usually spawned repeatedly over a time period exceeding the expected 21-day duration of a spermatogenic cycle ([50]; i.e., every two weeks for up to eight weeks). The result of repeatable sperm morphology, despite a change in environmental conditions between the field and the lab, suggests that differences between males are intrinsic properties of those males and not dominated by environmental effects. Furthermore, consistency in sperm morphology between 2006 and 2007 samples, despite differences in laboratory conditions of temperature and light regime, lend additional evidence to this conclusion. Understanding how size in sperm cells is controlled between males, and how intra-individual variance is limited will require a closer look at the molecular mechanisms controlling spermatogenesis in this system.

Ejaculate and sperm traits in fish have been shown to adjust in response to change in social status and therefore risk of sperm competition [51]. In addition, male fowl exhibit phenotypic plasticity in ejaculate size [52-54] and sperm velocity [55] under varying risk of sperm competition and depending on female number, quality and mating history. It is possible that similar plasticity exists in sea urchins, perhaps in response to population density, but because the captive environment was held constant for the duration of the study, we were unable to assess any plasticity in sperm morphology relative to sperm competition risk (e.g., density).

### Variation among males

We found highly significant variation among individuals within all three populations for all sperm traits (Table 1; head length shown in Figure 1B) with the exception of midpiece area in the East Atlantic population (*F*_5,130 _= 0.76, *P *= 0.58). Significant correlations among males were also found between head length and width (*r *= -0.379, *P *= 0.0006), head length and axoneme length (*r *= -0.349, *P *= 0.0016), axoneme length and total length (*r *= 0.954, *P *< 0.0001), and head width and midpiece area (*r *= 0.565, *P *< 0.0001; Table 1).

An evolutionary process separate from that acting among males may influence the developmental system that produces sperm within a male. Significant differences in sperm morphological variation among males coupled with high repeatability provide strong support for the hypothesis that males control sperm morphology around an individually based mean. Yet, there is some variation within a spawning, and the CVs of sperm size within a male are comparable to those among males within a population.

### Variation among populations

Population means were different for all sperm traits except total length (*F*_2,76 _= 2.9, *P *= 0.06), including head length, head width, axoneme length and midpiece area (Table 1). Sperm heads in the Pacific are long (mean head length = 6.95 μm, SD = 0.29) and narrow (mean head width = 1.60 μm, SD = 0.07) but in the East Atlantic, they are short (mean head length = 5.74 μm, SD = 0.44) and wide (mean head width = 1.94 μm, SD = 0.12), with the West Atlantic having an intermediate head shape (mean head length = 6.22, SD = 0.36; mean head width = 1.64, SD = 0.13; Table 3). Axoneme length was longest in the Pacific and shortest in the East Atlantic and negatively correlated with head length (Table 1), resulting in an overall equality of total length in all three populations. Head length was the only trait with significantly different means for all populations, according to a Tukey test (Table 4). Head width was significantly different only in the East Atlantic population, and axoneme length increased from west to east, with the West Atlantic not significantly different from the Pacific or East Atlantic populations. Midpiece area was distinct only for the West Atlantic population (Table 4).

**Table 3:** Population means and SD, average divergence among populations of sperm traits. Divergence expressed in units of average within-population phenotypic standard deviation (SD).

	Pacific		West Atlantic		East Atlantic			
	Mean	SD	Mean	SD	Mean	SD	Avg SD	Divergence
HL	6.95	0.29	6.22	0.36	5.74	0.44	0.37	2.21
HW	1.6	0.07	1.64	0.13	1.94	0.12	0.10	2.16
AL	42.4	1.20	44.02	1.79	45.02	0.75	1.24	1.40
TOTAL	49.32	1.09	50.31	1.80	50.77	0.78	1.22	0.79
MA	1.67	1.18	2.12	1.17	1.75	1.03	1.13	0.27

**Table 4:** Results of Tukey test (d.f. 3, 76) for multiple comparisons of population means.

	P	WA	EA
HL	A	B	C
HW	A	A	B
AL	A	AB	B
TOTAL	A	A	A
MA	A	B	A

Significantly different groups (*P *< 0.01) designated with different letters (A, B, C). For example, all three populations are different for HL, but none are different for TOTAL. Trait abbreviations as in Table 1.

**Figure 2:**
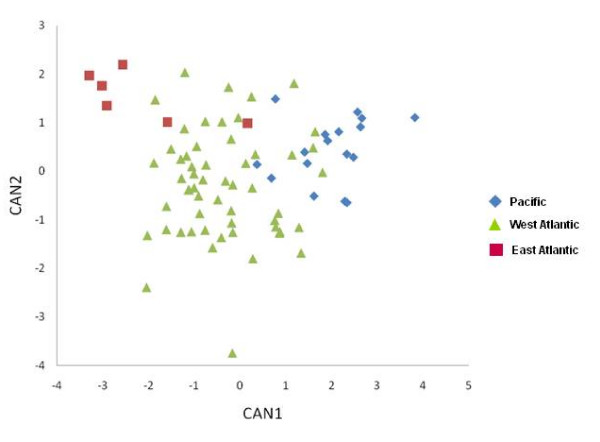
**Overall morphological variation among populations**. Scatterplot of CAN1 on CAN2 from multivariate canonical discriminant analysis. Blue diamonds are Pacific individuals, green triangles West Atlantic, and red squares East Atlantic.

Multivariate canonical discriminant analysis found significant among-population variation for overall sperm morphology, with two canonical variables (Figure 2; Wilks' lambda = 10.68, *P *< 0.0001). CAN1 accounted for 75.7% of the variation and had highest raw canonical coefficients for head length, axoneme length, and total length (Additional file 1). Pairwise Mahalanobis distances among the three populations showed Pacific sperm morphology to be most divergent from the East Atlantic, with the West Atlantic showing approximately equal divergence from both the Pacific and the East Atlantic (Additional file 2).

Population trait means did not differ in the West Atlantic between 2006 and 2007 for all traits except head width (Table 1), suggesting that the population means for most sperm traits are stable over at least two reproductive seasons. Further research is needed to determine longer-term persistence of measured population means for this and the other populations.

To date, investigations of variation in sperm morphology among populations are limited, but population-level variation has been found across a wide range of taxa. A study of two closely related *Drosophila *species found significant differences in sperm length among individuals and populations in both species [56]. A study in *Drosophila subobscura *found population differences in sperm head length but not total length [57], and [58] found significant variation among males in total length within and among four populations of a frog.

**Figure 3:**
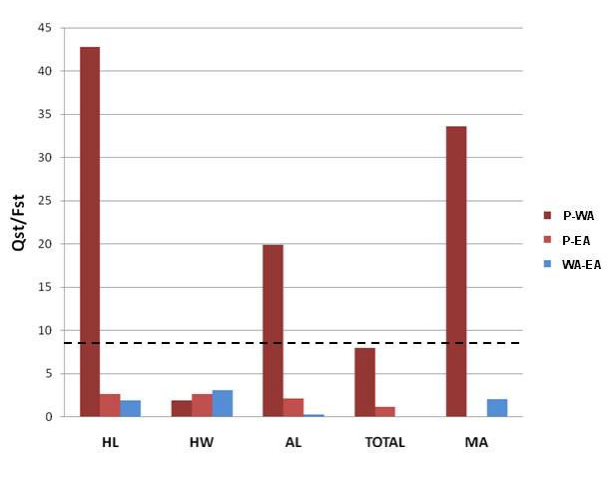
**Pairwise *Q*_ST_/*F*_ST _for all population pairs and all sperm traits**. Atlantic-Pacific comparisons are in different shades of red. Pacific-West Atlantic (P-WA) in dark red, Pacific-East Atlantic (P-EA) comparison in pink, and West-East Atlantic (WA-EA) comparison in blue. Head length (HL), head width (HW), axoneme length (AL), total length (TOTAL), midpiece area (MA). Dashed line represents the lower limit for statistically significant *Q*_ST_/*F*_ST _(α = 0.05) after Bonferroni correction.

In *S. droebachiensis*, substantial sperm differences among populations could be derived from a number of causes. The Atlantic populations probably originated from an invasion from the Pacific 3.5 million years ago with the opening of the Bering Strait [59]. Since that time, the western Atlantic population has received substantial influx from the Pacific [60], while the eastern Atlantic population has remained largely separated [61]. This demographic history is reflected in its population genetics [62], with an *F*_ST _between the Atlantic populations of 0.204 and between the western Atlantic and Pacific of 0.014. In contrast, the two Atlantic populations are more similar to each other in overall sperm morphology than they are to the Pacific (Table 4). In particular, *Q*_ST _divergence between the Pacific and West Atlantic exceeds their pairwise *F*_ST _by a factor of 47, more than an order of magnitude higher than for the other population comparisons and traits (Figure 3).

Ecological differences among populations do a better job than genetic relationships at explaining the pattern of variation in sperm head length. In the Pacific, *S. droebachiensis *is embedded in a complex community that includes 2–4 congeners, some of which may act as competitors for resources. In these Pacific habitats, *S. droebachiensis *is currently at lower population densities than the shallow water congeners, *S. purpuratus *and *S. franciscanus *[34], and has been characterized as a species that is sperm-limited in its spawning events [34,63]. In the Atlantic, on the other hand, *S. droebachiensis *can form dense stands of urchins that are capable of monopolizing the sea floor and stripping the habitat of macroalgae [64,65]. In such areas of high population density, sperm competition will be stronger than in most Pacific areas. The ecological similarity of the two Atlantic populations is more closely associated with the morphological similarity of sperm head length between these two populations relative to the Pacific. Increasing the sample size of the east Atlantic dataset and further study of historical population sizes may provide a clearer pattern of any association between sperm head morphology and population density among the study populations. In addition, experimental fertilization trials will be needed to test for any functional significance of these differences.

Evolutionary processes responsible for the observed sperm morphological variation among males may be illuminated by examining similar patterns in other male reproductive traits. In particular, the gamete recognition protein bindin shows strong differences between species as well as between some populations ([66-68] but also has high levels of polymorphism among individuals. Bindin has been shown to be evolving under balancing selection, mediated by sex-dependent, frequency-dependent, and density-dependent selection in the red sea urchin (*S. franciscanus*) [69,70], and the context-dependent nature of bindin's fitness effects may explain both its rapid evolution and high allelic diversity [71]. If balancing selection is also acting on sperm morphology, we might see high morphological diversity within populations (as shown here). We would also expect fitness effects of sperm traits to be context dependent. These different contexts may be characterized by variation in population density (and therefore risk of sperm competition), egg morphology and/or turbulence due to wave action, but further experimental evidence is needed.

Alternatively, sperm morphological traits may be evolving neutrally among males within populations of *S. droebachiensis*. Determination that intraspecific variation is under selection will require further comparisons with other species that occur at higher abundances (e.g., *S. purpuratus *and *S. franciscanus*) and an understanding of the dynamics of sperm precedence in males with different sperm head sizes.

### Inferring selection by comparing *Q*_ST _and *F*_ST_

Heterozygosities and tests of Hardy-Weinberg equilibrium for the East Atlantic and Pacific populations are as reported in [62] for the Vestfjorden, Norway and San Juan Channel, Washington populations, respectively. The West Atlantic population had a heterozygote deficit at two loci (Loc76 and Loc63) and between 16 and 23 alleles per locus with an average of 18.75. Global *F*_ST _was 0.159, with pairwise *F*_ST_'s of 0.014 between the Pacific and West Atlantic, 0.318 between the Pacific and East Atlantic, and 0.203 between the two Atlantic populations.

Quantitative genetic divergence among populations exceeded microsatellite divergence for most sperm traits. Average *Q*_ST _for the sperm traits was 0.41, with a standard error of 0.10, as compared with *F*_ST _of 0.159. ANOVAs of trait divergence from which variance components were obtained for calculating *Q*_ST _were significant for all traits except total length. *Q*_ST_'s for head length (*Q*_ST _= 0.57; *P *= 0.028), head width (*Q*_ST _= 0.65; *P *= 0.017), and midpiece area (*Q*_ST _= 0.50; *P *= 0.043) were significantly higher than *F*_ST_, based on tail probabilities of *Q*_ST _on a chi-squared *F*_ST _distribution ([72]; Table 2), although these results are not significant after Bonferroni correction.

Calculation of population pairwise *Q*_ST _and *F*_ST _shows that marginal signatures of selection at the global level are driven entirely by highly significant quantitative trait divergence between the Pacific and West Atlantic. Sperm head length (*Q*_ST_/*F*_ST _= 42.76; *P*_*X*_^2 ^= 6.20 × 10^-11^), axoneme length (*Q*_ST_/*F*_ST _= 19.87; *P*_*X*_^2 ^= 8.28 × 10^-6^) and midpiece area (*Q*_ST_/*F*_ST _= 33.62; *P*_*X*_^2 ^= 6.68 × 10^-9^), are most divergent, in comparison with a pairwise *F*_ST _of 0.0136 (Figure 3).

We used repeatability of sperm traits as an estimate of the upper limit of broad-sense heritability in our calculations of *Q*_ST_; the actual narrow-sense heritability for the sperm traits may be substantially lower. However, heritability of sperm morphometrics has been measured directly in a number of other species and has been found to be generally high. For example, heritability of sperm head length has been estimated to be 0.48 in zebra finches [73] and 0.72 in rabbits [74], comparable to our upper limit estimate of 0.77.

**Figure 4:**
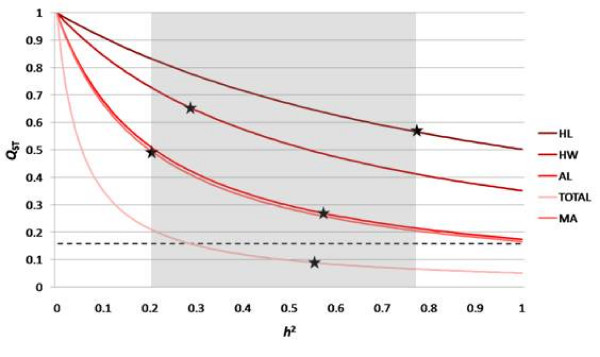
***Q*_ST _for sperm traits over all possible values of *h*^2 ^from 0 to 1**. Stars indicate the values estimated in this study for sperm traits. Dashed line represents global *F*_ST _= 0.159. Trait abbreviations are as in Figure 3. Shaded gray area represents the range of repeatabilities estimated in this study.

Nevertheless, the use of repeatability in the calculation of *Q*_ST _requires discussion of two key points. First, because repeatability is an upper limit on heritability, these *Q*_ST _estimates represent their lower limit given the observed phenotypic population differentiation and are therefore not expected to further approach *F*_ST_. Our estimates of *Q*_ST _are thus very conservative, because heritability appears in the denominator of the *Q*_ST _calculation and therefore has an inverse relationship with *Q*_ST _(Figure 4). Using repeatability as an upper limit on heritability means that our *Q*_ST _estimates represent a lower bound. Over all possible values of *h*^2^, *Q*_ST _remains above *F*_ST _for all sperm traits except total length, though both axoneme length and midpiece area approach *F*_ST _as *h*^2 ^reaches 1 (Figure 4).

Second, *F*_ST _and *Q*_ST _normally follow a chi-squared distribution ([72]), but *Q*_ST _as estimated using repeatability, which estimates broad-sense heritability, no longer follows the same theoretical rules as a *Q*_ST _estimated with an estimate of narrow-sense heritability. We can still calculate the tail probability of a point estimate of *Q*_ST _on our chi-squared distribution of *F*_ST_, but we are unable to make any more rigorous statistical tests that take the distribution of *Q*_ST _into account. This limitation also applies to the analysis of pairwise *Q*_ST _and *F*_ST_.

As a more general precautionary note, our results, and those of many *Q*_ST_-*F*_ST _comparisons, should be interpreted with a degree of caution. First, the chi-squared approximation of the *F*_ST _distribution holds best when *F*_ST _is less than 0.1 [72]. While our observed global *F*_ST _of 0.159 may still reasonably follow a chi-squared distribution, the predictive capability of this model is diminished. Furthermore, an excess of *Q*_ST _over *F*_ST _may be obtained from selection acting not on the traits under study but on genetically correlated traits [72]. Finally, both *F*_ST _and *Q*_ST _are historical signatures of genetic divergence accumulated over time and are therefore unable to identify contemporary selection. Indeed, a result of *Q*_ST _> *F*_ST _may result from selection or a response to past selection; these two possibilities cannot be differentiated by this analysis. Estimates of contemporary selection in this system may be especially useful, if evolution of sperm morphology is influenced by conditions controlling sperm competition that change on an ecological timescale. In short, the comparison of *Q*_ST _and *F*_ST _is a relatively blunt tool in evolutionary biology that can allow us to rule out genetic drift as a mechanism for quantitative trait differentiation and identify interesting traits for further study. The implications of directional selection derived here are preliminary at best and require more in-depth and rigorous analyses of selection for validation and detailed characterization.

### Head length and total length

Of all sperm traits examined, head length was the only measurement to show stability over time within an individual as well as within a population, strong variation among males within all populations, and strong differentiation among all populations. Head length also had a strong signature of directional selection both at the global and population pairwise levels. Patterns of head elongation among sea urchin species are associated with the evolution of large eggs and direct development [40] (also see [42]), suggesting a mechanical role for sperm head shape in penetrating the egg's thick jelly coat. In other externally fertilizing taxa, phylogenetic comparisons show more ambiguity in an association between sperm morphology and egg morphology, with a positive relationship found in frogs [7], but not in fish [4,6]. In addition, sperm head shape, along with relative flagellum length, is positively correlated with risk of sperm competition across frog species [7]. The only evidence for a direct correlation between function and morphology within a species with flagellated sperm has been found in an internal fertilizer, the red deer [75]. Velocity is known to predict fertilization success in a wide variety of taxa [21,76-78], including sea urchins [79], and head shape in broadcast spawners could play a role in swimming speed under different biomechanical conditions (e.g., turbulence, water velocity).

Total sperm length, on the other hand, showed no significant divergence or directional selection among populations. Other studies using phylogenetic contrasts and direct experiments also fail to find a significant relationship between indices of selection, such as sperm competition, with sperm total length [18-23]. This lack of selective signal, may be due to opposing evolutionary forces acting on different subcomponents of sperm, such as head length and axomenal length that comprise total length. The negative correlation that we found between these two measurements (Table 1) underscores the importance of measuring subcomponents of sperm in addition to total length.

## Conclusion

We found highly significant differences in sperm morphology among individuals within all populations for almost all traits, as well as strong population-level differentiation for head length, axoneme length, and midpiece area. At the same time, most traits showed low variation among multiple spawnings within a male. These results suggest that sperm morphology tends to be developmentally stable over time, while evolutionary forces are maintaining high levels of variation among individuals and populations of *S. droebachiensis*. Comparison of *Q*_ST _with *F*_ST _suggests that directional selection may be acting among populations for overall sperm morphology, especially between the Pacific and West Atlantic populations, in which sperm evolution has greatly outpaced neutral genetic divergence.

Directional selection for different means between populations of broadcast spawners could be driven by a wide variety of ecological differences, such as population density as a result of community structure. Individual males also differ in sperm size within populations, suggesting that selection on sperm traits is not only directional but may also involve balancing selection for traits that are most successful in different environments. Males from many species show this pattern in reproductive traits, suggesting that balancing selection, if acting, is not mating system-specific. It is also possible that variance between populations and species is under selection but that variance between males is drifting neutrally. The underlying genetic and developmental architecture that leads to variance between males in sperm morphology are largely unknown but represent a key area of research in elucidating the evolution of complex morphological phenotypes.

## Methods

### Obtaining and preparing samples

We examined sperm morphology in green sea urchins (*Strongylocentrotus droebachiensis*) from three populations, from the eastern Pacific (Friday Harbor, Washington, USA), West Atlantic (Woods Hole, Massachusetts, USA) and East Atlantic (Bødo, Norway). Individuals were obtained from the Pacific and West Atlantic from January to April 2006 and from the West Atlantic from February to April 2007. Sperm samples from the East Atlantic population were kindly provided by Nils Hagen in March 2006.

In 2006, adult *S. droebachiensis *were maintained at 14°C in outdoor seawater tanks under the local natural light regime at Hopkins Marine Station in Pacific Grove, California, USA and fed giant kelp (*Macrocystis pyrifera*) *ad libitum*. These individuals were reproductively active until late April. In 2007, a different cohort of adults was kept in an indoor seawater facility maintained at 8°C under a daily cycle of 13 hours of light and 11 hours of darkness. These individuals were also fed kelp *ad libitum *and were reproductively active through mid-June. Comparison of sample means between the different treatment conditions of 2006 and 2007 were done for the West Atlantic population using a t-test (see Data analysis section below).

Sea urchins were induced to spawn by injecting 0.55 M KCl. Most individuals were spawning on arrival after shipment, allowing a baseline measurement of sperm morphology before placement in common tanks. Dry sperm was collected off gonopores using a pipettor with a wide-bore tip, diluted 1:50 or 1:100 in filtered sea water (FSW), and fixed in a final concentration of 1% paraformaldehyde and 9.25% FSW.

### Sperm fixation, microscopy and morphometrics

Ten μl of fixed sperm were pipetted on a slide, and a cover slip was applied and sealed with nail polish. Individual spermatozoa were visualized using differential interference light (DIC) microscopy with a Zeiss Axioplan DIC microscope at 250× to 1000× magnification. Digital micrographs were taken using an Olympus E330, E995 or E4500 digital camera. Measurements on images were obtained using ImageJ software (available at http://rsb.info.nih.gov/ij/) and converted from units of pixels to microns based on a scale specific to the focal length of the camera and the ocular magnification. Scales were calculated using a stage micrometer (SPI Supplies, ser. no. CS2397).

We measured five sperm traits: head length, head width, axoneme length, total length, and midpiece area (Additional file 3). All spermatozoa chosen for measurement appeared normal with a visible endpiece to ensure presence of the entire axoneme. Total length did not include the endpiece. In 2006, we measured these traits for 15 males from the eastern Pacific, 22 males from the West Atlantic and six males from the East Atlantic (19–27 spermatozoa each). We also measured 33 males from the West Atlantic in 2007 (ten spermatozoa each) to assess annual variation in that population. All sperm measurements were repeatable across two measurement events (Table 1) at the *α *= 0.05 level after Bonferroni correction for multiple comparisons [80].

### Data analysis

Normality of data by individuals and populations were evaluated visually using box plots and scatter plots generated in SAS v. 9.1 (SAS Institute 2002), and only midpiece area data were log-transformed. All statistical analyses described below were performed in SAS, and Bonferroni correction was applied for each analysis. Repeatability of sperm measurements across two separate measurement events was determined for five males (ten spermatozoa each) from all three populations, using repeated measures ANOVA. We also spawned 15 males from the west Atlantic population (from both 2006 and 2007) every two weeks a total of two to five times and measured ten to 25 sperm from each spawning event to evaluate individual variation through time. These data were also analyzed using repeated measures ANOVA. We estimated correlations between pairwise individual trait means across the entire dataset using Pearson correlation coefficients. We also estimated coefficients of variation (CV) within and among males for all populations.

We examined the 2006 and 2007 spawning seasons in the west Atlantic population for differences in sperm morphology using a two-sample t-test and determined that only head width was significantly smaller in 2007 than in 2006 (Table 1). For this trait, subsequent analyses considered the 2006 and 2007 samples separately for the West Atlantic population. We used ANOVA to examine differences among individuals for each population separately as well as among populations, using individual means. Post-hoc multiple comparisons tests for differences of population means were performed using Tukey tests for all sperm traits. In order to account for population differentiation of multivariate sperm morphology, we performed a canonical discriminant analysis (PROC CANDISC; SAS v. 9.1), which asks if sperm from all populations are morphologically indistinguishable overall.

We tested the hypothesis that the amount of divergence in sperm traits among populations was significantly higher than we would expect for neutral variation, indicating directional selection for different trait means among populations. This test of selection was performed for each sperm trait by comparing quantitative genetic divergence or *Q*_ST _with *F*_ST _at neutral microsatellite markers. *Q*_ST _was estimated from descriptive components of variance obtained by analysis of variance, computed using RANDOM in PROC GLM in SAS and Type III sums of squares accounting for unbalanced design. *Q*_ST _was calculated as $Q_{\text{ST}}=\frac{V_{GB}}{V_{GB}+2V_{GW}}$[44], where *V*_*GB *_is the among-population variance for quantitative traits, and *V*_*GW *_is the average within-population genetic variance.*V*_*GW*_, in turn, was computed as the product of the trait heritability (*h*^2^) and the within-population component of variance (*V*_*W*_): *V*_*GW *_= *h*^2^*V*_*W*_.

We derived our estimates of heritability for each sperm trait from our measurements of repeatability, calculated using morphometric data from multiple spawnings of the same male [81]. Repeatability was determined using variance components of among-spawnings ANOVA and represents the maximum value of the broad-sense heritability of a trait [82]. Heritability is inversely related to *Q*_ST _due to its position in the denominator of the *Q*_ST _equation. As a result, the estimates of *Q*_ST _calculated using repeatability are very conservative and represent a lower limit on possible *Q*_ST_'s over the range of heritability from 0 to 1, given the observed among-population trait divergence.

*F*_ST _at four neutral microsatellite markers was estimated using AMOVA in Arlequin v. 2.000 [83] from published [62] and unpublished data (J. Addison) for the San Juan Islands, Washington, USA (*n *= 41); Isle of Shoals, New Hampshire, USA (*n *= 144); and Vestfjorden, Norway (*n *= 79). All of these sites are geographically identical or proximate to those from which adults were obtained for the sperm variation data. While *F*_ST _was not estimated from the same individuals from which sperm measurements were taken, both the sperm morphometric and microsatellite datasets were derived from the same geographic populations. The West Atlantic population had the largest distance between sampling localities of the two datasets, but previous research has shown that the West Atlantic region experiences high levels of gene flow [62]. Therefore, we do not expect these two localities (Isle of Shoals, New Hampshire and Woods Hole, Massachusetts) to be significantly genetically distinct. We did not obtain a standard error for *F*_ST_, because jackknifing cannot be done over only three populations. Mitochondrial DNA has also been used to estimate *F*_ST _[84,85], but these genes represent a single locus that may not be evolving under neutrality [86] and so were not included in this analysis.

We tested the hypothesis that our estimates of *Q*_ST _were significantly different from the neutral model represented by *F*_ST_. Because *F*_ST _estimates can be highly variable among neutral loci, it is best to compare *Q*_ST _not to a mean *F*_ST _but to a distribution of possible *F*_ST_'s [72]. The distributions of neutral *F*_ST _and *Q*_ST _have been shown to follow a chi-squared distribution under a wide range of demographic scenarios [72]. As a result, we can compare the *Q*_ST_-to-*F*_ST _ratio to a chi-squared distribution with (*n*_demes _- 1) degrees of freedom, according to the statistic (*n*_demes _- 1)*Q*_ST_/*F*_ST _[72], where *n*_demes _is the number of demes. The p-value associated with this statistic gives the probability that the observed *Q*_ST _falls within the distribution of *F*_ST_. A significant p-value for a sperm trait would indicate that it has a low probability of being selectively neutral and a high probability of evolving under directional selection.

To test for an effect of low sample size in the East Atlantic population, we performed a Bartlett's test for homogeneity of variances among all populations for each sperm trait. None of the sperm traits examined showed significant differences in variance among the populations, suggesting that although the East Atlantic sample size is small, there was no associated increase in variance. Most of the statistical tests performed in this study are based on ANOVA, which assumes equal variance among samples. Thus, we do not feel that the small sample size of the East Atlantic population has compromised our results in any way.

## Authors' contributions

MM collected and analyzed data and drafted the manuscript. SP provided input on data analysis and the manuscript. Both authors read and approved the final manuscript.

## Supplementary Material

Additional file 1 Table of sperm trait correlations (r) above diagonal and P-values below diagonal. Click here for file

Additional file 2 Table of raw canonical coefficients of sperm traits for both canonical variables from canonical discriminant analysis. Click here for file

Additional file 3 Table of squared Mahalanobis distances from canonical discriminant analysis of overall sperm morphology between population pairs Click here for file

Additional file 4 Green sea urchin sperm (A) components and (B) traits measured. Click here for file
