# Supplementary material for: Intraspecific divergence in sperm morphology of the green sea urchin, Strongylocentrotus droebachiensis: implications for selection in broadcast spawners
Source: BMC Evol Biol. 2008 Oct 13;8:283. doi: 10.1186/1471-2148-8-283 (PMC2613923; doi:10.1186/1471-2148-8-283)
Supplement: Additional file 1 — Table of sperm trait correlations (r) above diagonal and P-values below diagonal. [file 1471-2148-8-283-S1.doc]

## Additional file 1 - Sperm trait correlations (*r*) above diagonal and *P*-values below diagonal.

|  | HL | HW | AL | TOTAL | MA |
| --- | --- | --- | --- | --- | --- |
| HL |  | -0.379 | -0.349 | -0.085 | 0.191 |
| HW | 0.0006 |  | 0.273 | 0.186 | 0.565 |
| AL | 0.0016 | 0.015 |  | 0.954 | 0.163 |
| TOTAL | 0.454 | 0.101 | <0.0001 |  | 0.234 |
| MA | 0.091 | <0.0001 | 0.15 | 0.038 |  |
