# Supplementary material for: Intraspecific divergence in sperm morphology of the green sea urchin, Strongylocentrotus droebachiensis: implications for selection in broadcast spawners
Source: BMC Evol Biol. 2008 Oct 13;8:283. doi: 10.1186/1471-2148-8-283 (PMC2613923; doi:10.1186/1471-2148-8-283)
Supplement: Additional file 2 — Table of raw canonical coefficients of sperm traits for both canonical variables from canonical discriminant analysis. [file 1471-2148-8-283-S2.doc]

## Additional file 2 - Raw canonical coefficients of sperm traits for both canonical variables from canonical discriminant analysis

CAN1, First canonical variable; CAN2, second canonical variable.

|  | CAN1 | CAN2 |
| --- | --- | --- |
| HL | 16.88 | 10.39 |
| HW | -6.929 | 10.84 |
| AL | 24.09 | 45.34 |
| TOTAL | -42.57 | -62.95 |
| MA | 1.052 | 0.769 |
