# Supplementary material for: Intraspecific divergence in sperm morphology of the green sea urchin, Strongylocentrotus droebachiensis: implications for selection in broadcast spawners
Source: BMC Evol Biol. 2008 Oct 13;8:283. doi: 10.1186/1471-2148-8-283 (PMC2613923; doi:10.1186/1471-2148-8-283)
Supplement: Additional file 3 — Table of squared Mahalanobis distances from canonical discriminant analysis of overall sperm morphology between population pairs [file 1471-2148-8-283-S3.doc]

## Additional file 3 - Squared Mahalanobis distances from canonical discriminant analysis of overall sperm morphology between population pairs

Squared Mahalanobis distances above diagonal and *P*-values below diagonal.

|  | Pacific | West Atlantic | East Atlantic |
| --- | --- | --- | --- |
| Pacific |  | 5.94 | 18.62 |
| West Atlantic | <0.0001 |  | 6.78 |
| East Atlantic | <0.0001 | <0.0001 |  |
