# Supplementary figures and images for: Intraspecific divergence in sperm morphology of the green sea urchin, Strongylocentrotus droebachiensis: implications for selection in broadcast spawners
Source: BMC Evol Biol. 2008 Oct 13;8:283. doi: 10.1186/1471-2148-8-283 (PMC2613923; doi:10.1186/1471-2148-8-283)

**Additional file 4**

(A)


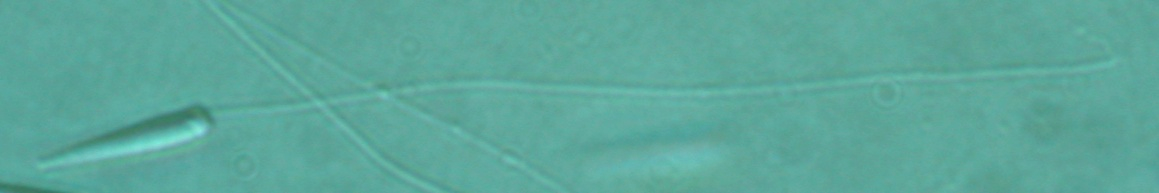


head

midpiece

axoneme

endpiece

(B)


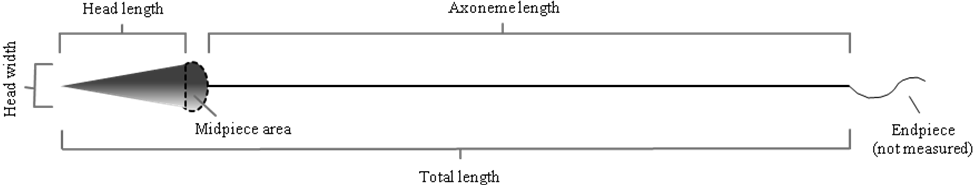

Supplement: Additional file 4 — Green sea urchin sperm (A) components and (B) traits measured. [file 1471-2148-8-283-S4.doc]
